# Food noise as a missing piece in obesity treatment: toward an integrated intervention framework

**DOI:** 10.1038/s41366-026-02185-1

**Published:** 2026-08-12

**Authors:** Abu Ansar Md Rizwan, Shannon Galyean

**Affiliations:** 1 Department of Nutritional Sciences, Texas Tech University, Lubbock, TX USA; 2 Nutrition and Metabolic Health Initiative, Texas Tech University, Lubbock, TX USA

**Keywords:** Weight management, Obesity

## Introduction

Obesity treatment has entered a new era with the widespread adoption of glucagon-like peptide-1 (GLP-1) receptor agonists and a striking patient-reported phenomenon, the silencing of persistent, intrusive food thoughts. Formally defined as “food noise”, persistent thoughts about food perceived as unwanted and/or dysphoric that may cause social, mental, or physical harm, this construct has moved from lay discourse into peer-reviewed science [1], is measurable through validated instruments, including the Ro Allison Indiana Dhurandhar Food Noise (RAID-FN) Inventory and the Food Noise Questionnaire, and resembles cognitive rumination more than physiological appetite [1, 2]. Yet food noise remains absent as an explicit therapeutic target from most obesity treatment frameworks. Recent consensus guidance calls for integrating nutritional and lifestyle strategies alongside GLP-1 therapy [3], and culinary medicine is emerging as a scalable behavioral intervention [4]. However, no prior framework has positioned food noise as the central unifying construct linking pharmacological, culinary-behavioral, and digital monitoring approaches. This Perspective argues that sustainable obesity management requires treating food noise not merely as a symptom to suppress, but as a measurable therapeutic target across neurobiological, culinary-nutritional, and cognitive-behavioral dimensions.

## The neurobiology of food noise and the GLP-1 window

GLP-1 receptors are expressed throughout appetite-regulating and reward-processing circuitry. Merkel et al. identified an endogenous nucleus tractus solitarius (NTS)-to-ventral tegmental area (VTA) circuit in which GLP-1-secreting neurons increase GABAergic inhibition, attenuating dopaminergic reward-seeking activity and providing a plausible neurobiological substrate for food noise reduction during semaglutide and tirzepatide treatment [5]. Cook (2026) further proposed that GLP-1 receptor agonists suppress default mode network hyperactivity, a mechanism through which mindfulness-based practices reduce food preoccupation [6]. This pharmacological window quiets neurobiological food noise and increases patients’ capacity to build behavioral skills. The critical problem is closure upon discontinuation, as food noise reliably returns within days to weeks, and the STEP 1 trial extension demonstrated approximately two-thirds weight regain within one year of semaglutide withdrawal [7]. Three converging problems expose the limits of pharmacotherapy alone: access inequity from cost, insurance gaps, and gastrointestinal side effects disproportionately affecting low-income and minority populations; failure to address environmental and emotional food noise drivers as patients re-enter processed food environments without behavioral skills [2]; and co-occurrence with anxiety, emotional eating, and binge eating requiring targeted psychological intervention.

## A three-component integrated framework

We propose that sustainable food noise management requires concurrent intervention across three domains, none of which is sufficient in isolation.

### Pharmacological component

GLP-1 receptor agonists remain the most potent tool for suppressing neurobiological food noise. This window should be framed as creating space for skill acquisition, not a permanent solution, and paired proactively with nutritional and behavioral intervention before discontinuation [7].

### Nutritional and culinary medicine components

Dietary patterns high in protein, fiber, and complex carbohydrates promote stable blood glucose and sustained satiety, whereas highly restrictive dieting can paradoxically intensify food-related cognitive preoccupation. Crucially, knowing what to eat is insufficient when patients lack the culinary self-efficacy to prepare it. Culinary medicine, an evidence-based field integrating cooking skills training with nutrition education [4], is uniquely positioned to address the environmental and behavioral dimensions of food noise that pharmacotherapy does not reach. A scoping review of 46 studies found that theoretical dietary knowledge alone consistently fails to produce sustained behavior change without practical culinary competence, identifying hands-on cooking as the strongest real-world facilitator [8]. A bi-center randomized controlled trial demonstrated that a remote culinary medicine program achieved 4.2% weight reduction sustained to 12 months with improvements in culinary self-efficacy and Mediterranean diet adherence comparable to naltrexone/bupropion [4]. The Mozaffarian et al. joint advisory identified culinary knowledge as essential for sustaining equitable GLP-1 outcomes [3]. Registered Dietitian Nutritionists (RDNs) are ideally positioned to deliver culinary medicine within the pharmacological window, utilizing GLP-1-mediated food noise suppression to build durable cooking skills and food literacy before discontinuation becomes necessary.

### Cognitive-behavioral component

Cognitive Behavioral Therapy restructures maladaptive food-related beliefs while mindfulness-based strategies target attentional mechanisms underlying food cue reactivity. A meta-analysis of 24 controlled trials found mindfulness-based interventions significantly reduced food craving intensity (Hedges’ *g* = 0.28; 95% CI 0.07–0.48) [9], and Cook proposed that mindfulness and GLP-1 pharmacotherapy share overlapping default mode network mechanisms, suggesting behavioral practice may partially sustain neurobiological benefits after discontinuation [6]. Mindfulness-Based Cognitive Therapy is particularly promising for patients whose food noise intersects with emotional dysregulation or disordered eating.

## The role of digital health and artificial intelligence in food noise monitoring

A critical gap is the absence of real-time food noise monitoring. While validated instruments provide cross-sectional measurement, food noise is dynamic, intensifying during discontinuation and dietary lapses [1, 2]. Ecological momentary assessment (EMA) via smartphone offers real-time capture of food-related cognitions [10], and integrating this with artificial intelligence (AI)-assisted dietary coaching platforms, already demonstrating accuracy in personalized dietary assessment and virtual cardiometabolic coaching [10], could identify food noise triggers, prompt cognitive-behavioral micro-interventions, and alert RDNs when food noise rebounds during dose reduction. This AI-augmented layer enables dynamic titration of all three framework components precisely when food noise intensifies.

## Conclusion and research priorities

Food noise represents a clinically meaningful but under-addressed dimension of obesity. This framework positions GLP-1 therapy as a neurobiological scaffold on which culinary and cognitive-behavioral skills are built, each addressing a distinct food noise dimension. Research priorities include randomized trials comparing time-limited GLP-1 therapy plus integrated intervention versus indefinite pharmacotherapy, gastrointestinal tolerability strategies to extend the pharmacological window, cross-cultural validation of food noise instruments [1], investigation of whether food noise reduction mediates cardiometabolic benefits of GLP-1 treatment, trials testing whether culinary medicine delivered during the GLP-1 window produces more durable behavior change than culinary medicine alone, and AI-assisted EMA for real-time food noise monitoring, a direction no prior study has yet examined. As food noise transitions from a lay descriptor to a formal clinical construct, the field has an opportunity to reframe obesity treatment, not merely as a disorder of energy balance, but as one in which the cognitive and emotional experience of food demands explicit therapeutic attention.

## References

[1] Dhurandhar EJ, Maki KC, Dhurandhar NV, Kyle TK, Yurkow S, Hawkins MAW, et al. Food noise: definition, measurement, and future research directions. Nutr Diabetes. 2025;15:30 10.1038/s41387-025-00382-x. PMC12238327 40628707

[2] Hayashi D, Edwards C, Emond JA, Gilbert-Diamond D, Butt M, Rigby A, et al. What is food noise? A conceptual model of food cue reactivity. Nutrients. 2023;15:4809 10.3390/nu15224809. PMC10674813 38004203

[3] Mozaffarian D, Agarwal M, Aggarwal M, Alexander L, Apovian CM, Bindlish S, et al. Nutritional priorities to support GLP-1 therapy for obesity: a joint Advisory from the American College of Lifestyle Medicine, the American Society for Nutrition, the Obesity Medicine Association, and The Obesity Society. Am J Clin Nutr. 2025;122:344–67. 10.1016/j.ajcnut.2025.04.023. PMC12612741 40450457

[4] Polak R, Budd MA, Finkelstein A, Goldsmith R, Goldstein R, Gray BE, et al. One-year weight loss following a remote culinary medicine program: a bi-center randomized controlled trial. Obesity. 2026;34:1045–55. 10.1002/OBY.70193 PMC13116008 41947527

[5] Merkel R, Hernandez NS, Weir V, Zhang Y, Caffrey A, Rich MT, et al. An endogenous GLP-1 circuit engages VTA GABA neurons to regulate mesolimbic dopamine neurons and attenuate cocaine seeking. Sci Adv. 2025;11. 10.1126/sciadv.adr5051 PMC11864183 40009667

[6] Cook G. Quieting “Food Noise”: how GLP-1s and mindfulness rewire the default mode network (DMN) and reward circuits. Cureus. 2026;18:100818. 10.7759/cureus.100818 PMC12770913 41502834

[7] Wilding JPH, Batterham RL, Davies M, Van Gaal LF, Kandler K, Konakli K, et al. Weight regain and cardiometabolic effects after withdrawal of semaglutide: the STEP 1 trial extension. Diabetes Obes Metab. 2022;24:1553–64. 10.1111/dom.14725. PMC9542252 35441470

[8] Casaña Mohedo J, Murillo-Llorente MT, Palau Ferre A, Faus M, Gómez Taylor B, Esteve Claramunt F, et al. Nutritional needs, dietary knowledge, and culinary skills in individuals with diabetes mellitus: a scoping review. Adv Nutr. 2026;17:100627. 10.1016/J.ADVNUT.2026.100627. PMC13141728 41951176

[9] Allameh SA, Mokhtari Z, Hosseini E, Askari G. Effects of mindfulness-based interventions on food craving in adults: a systematic review and meta-analysis of controlled clinical trials. BMC Psychology. 2025;13:1022. 10.1186/s40359-025-03307-6. PMC12465136 40999532

[10] Panayotova GG. Artificial intelligence in nutrition and dietetics: a comprehensive review of current research. Healthcare. 2025;13:2579 10.3390/healthcare13202579. PMC12563881 41154258

